# Vaccine-Like African Swine Fever Virus Strain in Domestic Pigs, Thailand, 2024

**DOI:** 10.3201/eid3202.251245

**Published:** 2026-02

**Authors:** Trong Tung Nguyen, Dhithya Venkateswaran, Anwesha Prakash, Quynh Anh Nguyen, Roypim Suntisukwattana, Anan Jongkaewwattana, Theeradej Thaweerattanasinp, Janya Saenboonrueng, Van Phan Le, Dachrit Nilubol

**Affiliations:** Swine Viral Evolution and Vaccine Development Research Unit, Chulalongkorn University, Bangkok, Thailand (T.T. Nguyen, D. Venkateswaran, A. Prakash, Q.A. Nguyen, R. Suntisukwattana, D. Nilubol); National Center for Genetic Engineering and Biotechnology, Pathum Thani, Thailand (A. Jongkaewwattana, T. Thaweerattanasinp, J. Saenboonrueng); College of Veterinary Medicine, Vietnam National University of Agriculture, Hanoi, Vietnam (V.P. Le)

**Keywords:** African swine fever, African swine fever virus, viruses, vaccines, pigs, Thailand, 2024

## Abstract

African swine fever virus genotype II is endemic in Thailand, typically causing acute disease. We investigated a vaccine-like strain, characterized by 6 multigene family gene deletions, from nonvaccinated herds. We found this strain was associated with chronic disease in pigs.

African swine fever (ASF) is a fatal hemorrhagic disease of pigs, caused by African swine fever virus (ASFV), a complex DNA virus in the Asfarviridae family (*1*). Researchers first identified ASF in Kenya in 1921, and subsequent reports identified 24 genotypes in Africa on the basis of nucleotide variations within the partial B646L gen (*2*,*3*). Reports in the medical literature confirm incidence of only ASFV genotype I and genotype II outside Africa. 

In 2018, researchers identified ASFV genotype II in China (*4*), and it rapidly spread across Asia within a few months. Since then, the situation in Asia has shifted from an epidemic to an endemic stage, with the highly virulent genotype II strain causing peracute, acute, and subacute disease. Recent research suggests the emergence of more genetically diverse ASFV variants, including chronic disease–associated genotype I, highly virulent recombinants of genotypes I and II, and naturally and artificially attenuated strains in domestic pigs in China and Vietnam (*5*–*7*). 

Thailand health authorities officially reported ASFV in Thailand in 2022 (*8*), and the strain was genetically identical to the strain first reported in China and Vietnam. Currently, ASF cases in Thailand involve patients with chronic symptoms and low mortality rates, suggesting the emergence of low-virulent strains. We conducted a survey of ASFV from recent outbreaks in Thailand, employing whole-genome sequencing to investigate the underlying causes.

Veterinary clinicians reported suspected disease in pigs from 2 herds located in the western region of Thailand, ≈500 miles apart, all displaying clinical signs related to chronic forms of ASF: chronic respiratory disease, joint swelling, slow weight gain, and sporadic deaths. Both herds housed only finishing pigs, operating on an all-in/all-out basis, and pigs were not vaccinated with any types of ASF vaccines. 

We submitted 25 blood and organ samples from ASF-suspected pigs to a Biosafety Level 3 laboratory at the National Center for Genetic Engineering and Biotechnology (Thailand Science Park, Pathum Thani, Thailand). We extracted viral DNA from samples following the protocol of the DNeasy blood and tissue kit (QIAGEN, https://www.qiagen.com). We detected ASFV by real-time PCR targeting the B646L gene, according to the World Health Organisation for Animal Health’s International Office of Epizootics manual (*9*). We performed whole-genome sequencing on the Illumina NovaSeq X platform (Illumina, https://www.illumina.com), generating 151 bp paired-end reads. We analyzed raw sequences according to methods described in a previous study (*8*). We used FastQC v0.74 to assess the raw data quality and removed adapters using BBDuk version 38.84. We aligned high-quality reads with Sscrofa11.1 (GCA_000003025.6) by Bowtie2 (https://bowtie-bio.sourceforge.net/bowtie2/index.shtml), eliminating >95% of host-derived contamination. We assembled cleaned reads de novo using SPAdes version 4.2.0 (https://github.com/ablab/spades/releases/tag/v4.2.0). We then mapped reads back to the assembled genome using Burrow-Wheeler Aligner-MEM (https://janis.readthedocs.io/en/latest/tools/bioinformatics/bwa/bwamem.html) and calculated coverage depth with Samtools version 1.21 (https://sourceforge.net/projects/samtools) to confirm average coverage. We analyzed the assembled genomes by BLASTn (https://blast.ncbi.nlm.nih.gov), choosing the closest match as the reference genome. We aligned 32 ASFV genomes from GenBank with the newly determined sequences using MAFFT version 7.526 (https://mafft.cbrc.jp/alignment/software). Finally, we constructed a phylogenetic tree in MEGA11 (https://www.megasoftware.net) with the neighbor-joining method and 1,000 bootstrap replicates.

We detected ASFV in 18 of 25 samples; cycle threshold (Ct) values ranged from 19.82 to 33.83. We performed whole-genome sequencing on samples with the lowest Ct from each herd,(Ct 19.82 for sample TH1_24/RB and Ct 21.25 for sample TH2_24/RB). We analyzed 2 completely sequenced ASFV genomes, which exhibited 38.5% guanine-cytosine content and average coverages of ≈163× and >96% breadth of 10× coverage. We deposited raw sequencing reads in the National Center for Biotechnology Information Sequence Read Archive (https://www.ncbi.nlm.nih.gov/sra; BioProject PRJNA1344271). We submitted the genomes to GenBank (accession nos. PX119974 and PX11995) and analyzed them in comparison with the Georgia 2007/1 strain (accession no. FR682468). Both genomes revealed the deletion of 6 genes in the multigene family (MGF) region (MGF 505-1R, MGF 360-12L, MGF 360-13L, MGF 360-14L, MGF 505-2R, and MGF 505-3R) and 2,348 bp of an *Escherichia coli* GusA gene (GUS) inserted at the deletion site (Figure 1). This deletion pattern was like a live-attenuated vaccine strain (ASFV-G-ΔMGF) and a field-attenuated isolate (ASFV-GUS-Vietnam) described in previous studies (*6*,*10*). Phylogenetic analysis based on full-length genome indicated that the 2 isolates belonged to genotype II; however, the isolates were genetically distinct from the genotype II variant responsible for the first outbreak in Thailand (Figure 2). The 2 variants contained a total of 15 mutations throughout the genome, mostly silent and in noncoding regions, when compared with the Georgia 2007/1 strain. In addition, a 3-nucleotide insertion resulted in 1 additional amino acid in the MGF 110-10-L-MGF110-14L fusion protein.

**Figure 1:**
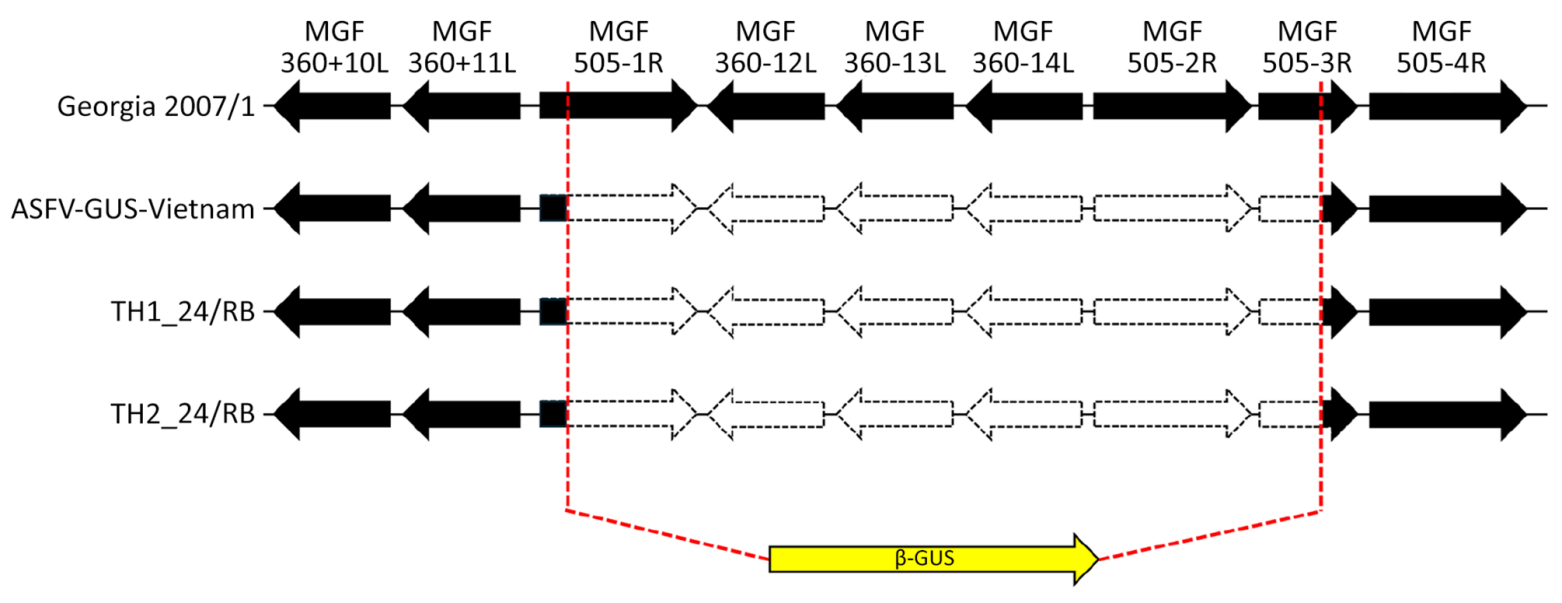
Schematic diagram from study of vaccine-like African swine fever virus strain in domestic pigs, Thailand, 2024, showing the deletion of the MGF gene region replaced by the β-GUS marker gene in the following strains: Georgia 2007/1, ASFV-GUS-Vietnam, and the 2 isolates from this study, TH1_24/RB and TH2_24/RB . Reference sequences obtained from GenBank. Deleted regions represented by dashed white arrows, intact genes by solid black arrows, and the inserted β-GUS gene by a yellow arrow. GUS, GusA gene; MGF, multigene family.

**Figure 2:**
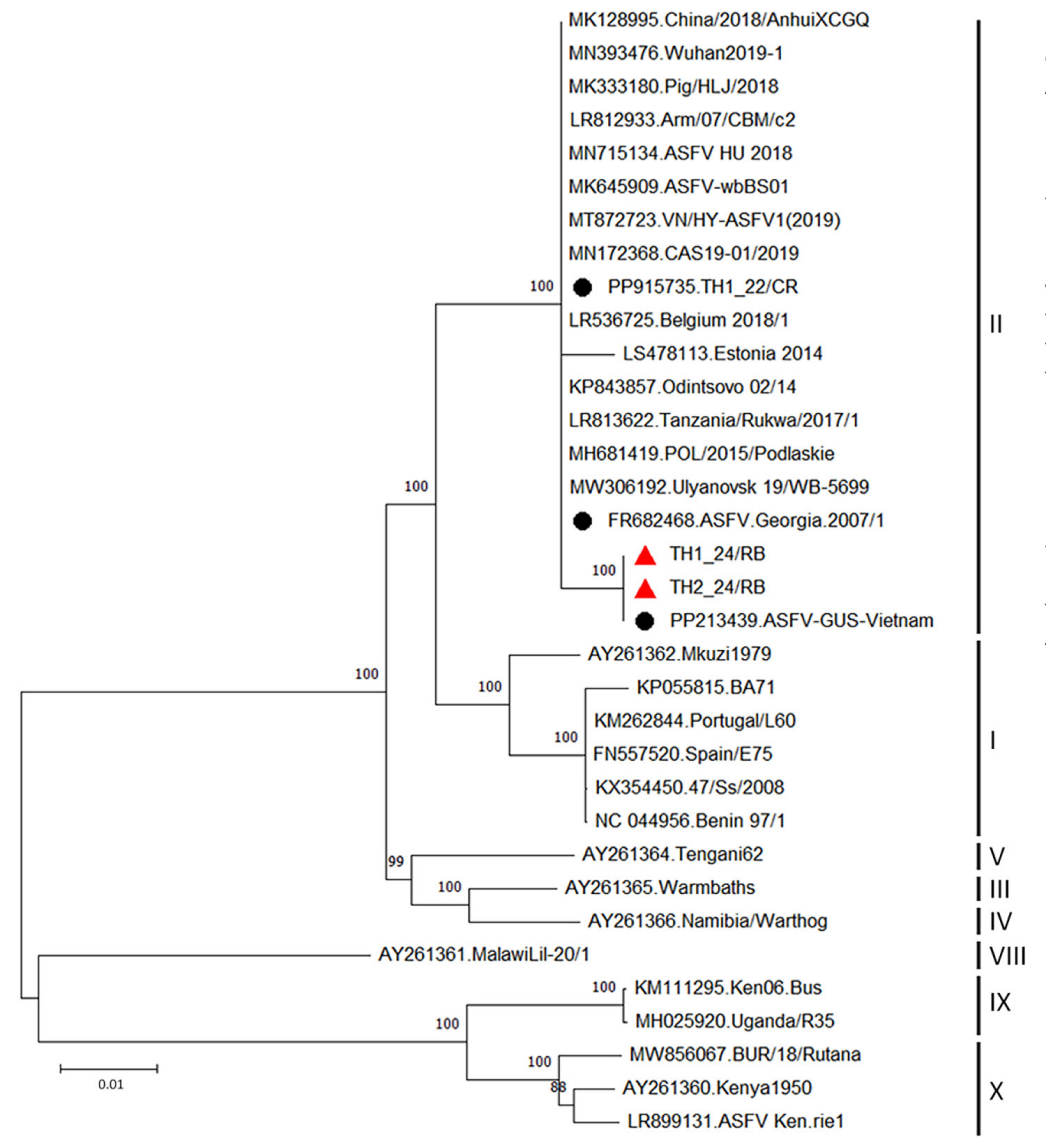
Phylogenetic tree based on whole-genome sequences of ASFV from a study of vaccine-like ASFV strain in domestic pigs, Thailand, 2024. Maximum-likelihood method and general time reversible model used in analyzing phylogenetic trees in MEGA 11 software (https://www.megasoftware.net). Red triangles indicate samples from this study; black circles indicate first ASFV strain in Thailand (TH1_22/CR), Georgia.2007/1, and ASFV-GUS-Vietnam. Genotypes are shown at right. Bootstrap analysis performed with 1,000 replicates; only bootstrap values >80 are shown. GenBank accessio numbers are provided for reference isolates. ASFV, African swine fever virus; GUS, GusA gene.

In conclusion, we characterized a vaccine-like genotype II strain, similar to ASFV-G-ΔMGF, detected in finishing pigs unvaccinated against ASFV in Thailand. The spread of such vaccine-like strains with MGF deletions in this region is of concern, and the origin of the strains remains unknown. Further genomic surveillance and epidemiologic tracing would assist in clarifying the route of introduction. Possible explanations include the unauthorized use of live attenuated vaccines or cross-border movement of pigs and pork products. 
